# Retraction Note: Tspan8 and Tspan8/CD151 knockout mice unravel the contribution of tumor and host exosomes to tumor progression

**DOI:** 10.1186/s13046-025-03284-z

**Published:** 2025-01-16

**Authors:** Kun Zhao, Zhe Wang, Thilo Hackert, Claudia Pitzer, Margot Zöller

**Affiliations:** 1 Pancreas Section, University Hospital of Surgery, Ruprecht-Karls-University, Heidelberg, Germany; 2 Interdisciplinary Neurobehavioral Core, Institute of Pharmacology, Ruprecht-Karls-University, Heidelberg, Germany; 3 Present Address: Department of Oncology, First Affiliated Hospital of Guangdong Pharmaceutical University, Guangdong, China

**Retraction Note: J Exp Clin Cancer Res 37, 312 (2018)**

10.1186/s13046-018-0961-6.

The Editor-in-Chief has retracted this article. Image integrity concerns were raised regarding Figs. 1e, 3c, and Fig. 6d. Further image concerns were found during analysis by the publisher in Figs. 2, 4, 7 and 8. The authors suggested a correction to these seven figures and figure legends in the manuscript. The ethics approval documents could not be provided upon request. The Editor-in-Chief determined that such extensive corrections could not be made without peer review, and that confidence has been lost in the data supporting the conclusions of the article.

Author Kun Zhao has stated on behalf of all authors that all authors disagree with this retraction.

